# Preoperative proximal tibial bone density, bone microarchitecture, and bone turnover are not associated with postoperative tibial component migration in cemented and cementless medial unicompartmental knee replacements: secondary analyses from a randomized controlled trial

**DOI:** 10.2340/17453674.2024.39917

**Published:** 2024-02-23

**Authors:** Mathias Alrø Fichtner BENDTSEN, Anders ODGAARD, Frank MADSEN, Sebastian Breddam MOSEGAARD, Jesper Skovhus THOMSEN, Ellen Margrethe HAUGE, Kjeld SØBALLE, Maiken STILLING

**Affiliations:** 1 Department of Clinical Medicine, Aarhus University, Aarhus; 2 AutoRSA Research Group, Orthopaedic Research Unit, Aarhus University Hospital, Aarhus; 3 Department of Orthopaedic Surgery, Rigshospitalet—Copenhagen University Hospital, Copenhagen; 4 Department of Clinical Medicine, University of Copenhagen, Copenhagen; 5 Department of Orthopaedic Surgery, Aarhus University Hospital, Aarhus; 6 Department of Biomedicine, Aarhus University, Aarhus; 7 Department of Rheumatology, Aarhus University Hospital, Aarhus, Denmark

## Abstract

**Background and purpose:**

Cementless arthroplasty fixation relies on early bone ingrowth and may be poor in patients with low proximal tibial bone density or abnormal bone turnover. We aimed first to describe the baseline bone properties in patients undergoing medial unicompartmental knee replacement (UKR), and second to investigate its association with cemented and cementless tibial component migration until 2 years.

**Methods:**

A subset investigation of 2 patient groups from a 3-armed randomized controlled trial was conducted. There were 26 cemented and 25 cementless medial UKRs with twin-pegged femoral components. Volumetric bone mineral density (vBMD) and microstructure of the excised medial tibial plateau were ascertained with µCT. Bone turnover was estimated using dynamic histomorphometry (eroded surface/bone surface = ES/BS, osteoid surface/bone surface = OS/BS, mineralizing surface/bone surface = MS/BS). Tibial component migration in 4 feature points was followed for 2 years with radiostereometry.

**Results:**

At the 2-year follow-up, the cementless tibial components migrated 0.38 mm (95% confidence interval [CI] 0.14–0.62) total translation more than the cemented components at the posterior feature point. The greatest migration in the cementless group was subsidence at the posterior feature point of 0.66 mm (CI 0.48–0.84) until 6 weeks, and from 3 months the components were stable. Cemented tibial components subsided very little. Between 1- and 2-year follow-ups, no cementless but 4 cemented tibial components revealed continuous migration.

OS/BS was half of the ES/BS. No µCT or histomorphometric parameters showed any clinically relevant correlation with tibial component migration at the posterior feature point for either cemented or cementless UKR at 6 weeks’ or 2 years’ follow-up after adjustment for age, BMI, and sex.

**Conclusion:**

Preoperative vBMD, bone turnover, and microstructure were not associated with postoperative tibial component migration of cemented and cementless medial UKR.

The use of medial unicompartmental knee replacement (UKR) is increasing for treatment of knee osteoarthritis [1]. The main reason for UKR revision is aseptic loosening of the prosthesis components [1] related to wear particles [2] or microfractures. The question is whether this can be explained by the baseline bone density, bone microarchitecture, or bone turnover [3]. Tibial component fixation can be determined by radiostereometric analysis (RSA), where a pattern of early migration predicts later revision surgery for aseptic loosening [4,5]. Baseline bone volumetric density (vBMD) and bone microarchitecture can be assessed with micro computed tomography (µCT) and bone turnover can be determined using histomorphometry of the tibial plateau removed during arthroplasty. In previous studies, the association between tibial component migration and preoperative areal bone mineral density (aBMD) assessed by DXA is ambiguous for both cemented UKR and cementless TKR [3,6].

Our aim was first to describe the baseline bone properties (volumetric bone density, bone microarchitecture, and bone turnover) in patients undergoing medial UKR, and second to investigate its association with tibial component migration until 2 years postoperatively.

## Methods

### Participants

**Figure 1:**
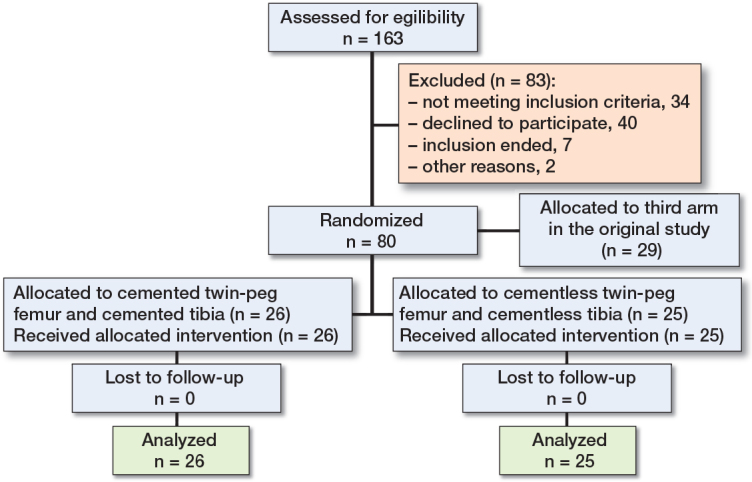
Consort flowchart. The complete dataset for the 26 patients (knees) in the cemented group and 25 patients (knees) in the cementless group was used for statistical comparisons.

A subset investigation was conducted of 2 patient groups from a 3-armed randomized controlled trial (n = 77) [7]. There were 26 cemented and 25 cementless medial UKRs with twin-pegged femoral components (Figure 1). All patients in the original 2 randomization arms were included in this subset investigation. The patients entered the study with 1 of their knees only. Patients were recruited at 2 Danish hospitals (Aarhus University Hospital and Vejle Hospital) between 2009 and 2011. Inclusion criteria were painful medial knee osteoarthritis, age >18 years, and informed consent. A detailed description of sub-study purposes is given at ClinicalTrials.gov (NCT00679120) and in previous publications [7,8] including the original 3-armed CONSORT flowchart.

The study is reported according to CONSORT guidelines.

### Prostheses

The prostheses were phase 3-alpha Oxford medial UKA with ArCom ultra-high molecular weight polyethylene-bearings and twin-pegged femoral components (Zimmer Biomet, Warsaw, IN, USA). In the cemented group, both the tibial and femoral components were fixed with Refobacin Bone Cement R (Zimmer Biomet, Warsaw, IN, USA). In the cementless group, the tibial and femoral components were coated with plasma-sprayed 750-µm-thick titanium and an additional coating of plasma-sprayed 55-µm-thick hydroxyapatite and inserted press-fit.

### Surgical procedure

The manufacturer’s recommendations for the surgical procedures were followed. The bone sample removed from the medial tibial plateau was marked on the anterior border and stored in 70% ethanol. Before implant component insertion, 6–8 1-mm tantalum beads (X-medics, Copenhagen, Denmark) were inserted in the proximal tibial bone with a bead gun (Kulkanon, Wennberg Finmek, Gunilse, Sweden) for later RSA.

### Bone samples and histomorphometry

**Figure 2:**
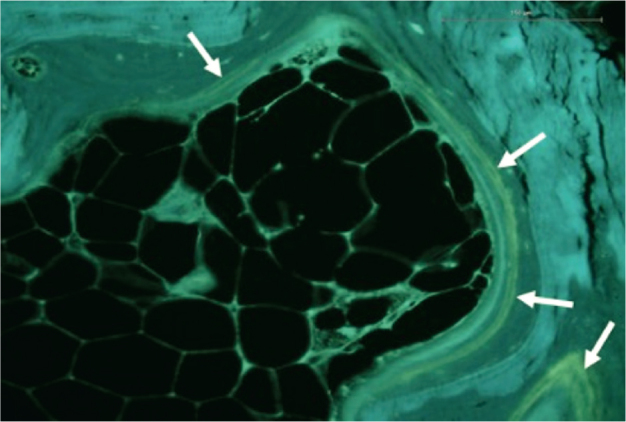
Unstained section of the proximal tibial bone assessed under ultraviolet light showing tetracycline double labels (white arrows). From the amount of tetracycline single labels and double labels the amount of mineralizing surfaces can be estimated (see text for detail).

**Figure 3:**
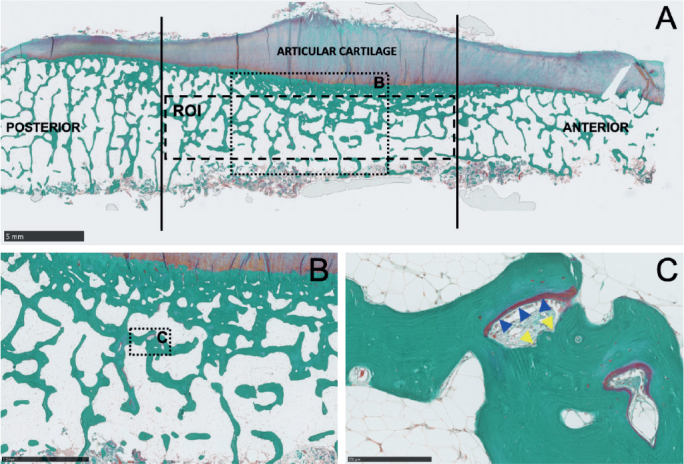
A. Masson–Goldner trichrome stained section of the proximal tibia showing the area analyzed with histomorphometry, bar = 5 mm. B. Magnification of B, bar = 2.5 mm. C. Magnification of C showing osteoid covered surface indicating bone formation (blue arrow heads) and erosive bone surfaces (yellow arrow heads), bar = 250 µm.

16 days prior to surgery, labelling of the bone for dynamic histomorphometry was initiated: 500 mg/day oral tetracycline (Tetracyklin “DAK,” Nycomed Danmark ApS, Roskilde, Denmark) for 2 days, 10-day pause, and 500 mg/day oral tetracycline for 2 days (Figure 2). Within 2 weeks after surgery, the bone samples were dehydrated in 70–96% ethanol, cleared in isopropanol and xylene, and embedded undecalcified in methyl methacrylate. 7-µm-thick sections were cut at 4 levels 100 µm apart (Reichart Jung, Polycut E microtome) and either stained with Masson–Goldner trichrome (Figure 3) for classification of the bone surfaces or left unstained for dynamic histomorphometry. Eroded surface (ES), osteoid surface (OS), and bone surface (BS) were estimated with a line grid using stereology software (version 6.6.1.2569, Visiopharm, Hørsholm, Denmark) on a PC connected to a microscope (BX 50, Olympus, Ballerup, Denmark), and ES/BS and OS/BS were computed [9]. Under ultraviolet light, intersections between the line grid and bone surfaces marked with either tetracycline single-labeled surface (sLS) or double-labeled surface (dLS) were counted on the unstained sections. Mineralizing surface per bone surface (MS/BS) was calculated as (sLS/2 + dLS)/BS. MS/OS is the ratio of mineralizing surfaces to osteoid surfaces and is equivalent to the fraction of osteoid seam life span during which mineralization occurs in the case of steady state [9]. Bone turnover is the amount of bone resorption and formation per unit time per unit bone volume [10]. Thus, bone formation rate (BFR)/BS is a proxy marker of bone turnover with ES/BS and OS/BS as alternative proxy bone turnover markers. The regions of interest (ROI) of the bone specimens spanned the 2 mm cancellous bone on the cut surface to avoid saw residue and bone tears in the histomorphometric analysis.

### Microcomputed tomography (µCT)

**Figure 4:**
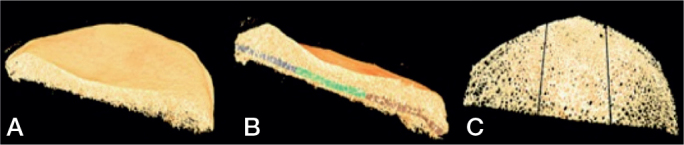
A. Superior view. B. Side view with the investigated volumes of interest (VOI) indicated: grey region = VOI-1 (anterior), green region = VOI-2 (central), and brown region = VOI-3 (posterior). C. The investigated volumes of interest.

The embedded bone samples underwent µCT (µCT35, Scanco Medical AG, Brüttisellen, Switzerland) in high resolution mode (1,000 projections/180°) using an isotropic voxel size of 18.5 µm, X-ray tube voltage of 70 kVp, current of 114 µA, and an integration time of 600 ms. Beam-hardening effects were reduced using a 0.5 mm aluminum filter. 3 1-mm-high volumes of interest (VOIs) were created using custom-made software [11] and placed parallel to and 1 mm proximal from the cut plane in order to exclude sawing residue (Figure 4). The VOIs covered the anterior (VOI-1), central (VOI-2), or posterior third (VOI-3) of the epiphyseal trabecular part of the bone sample (Figure 4). Thus, VOI-2 corresponded to the ROI used for histomorphometry. The 3D data sets were low-pass filtered using a Gaussian filter (σ = 1.3, support = 2) and segmented with a fixed threshold filter of 501 mg HA/cm^3^. For each VOI, standard microstructural parameters [12] were determined using the µCT scanner software (Image Processing Language, version 5.11, Scanco Medical AG, Brüttisellen, Switzerland). These included: vBMD, structure model index (SMI), trabecular thickness (Tb.Th), trabecular separation (Tb.Sp), and bone volume/tissue volume (BV/TV). 3-dimensional visualization was achieved using Amira 5.6 (FEI Visualization Science Group, Mérignac, France).

### Radiostereometric analysis

**Figure 5:**
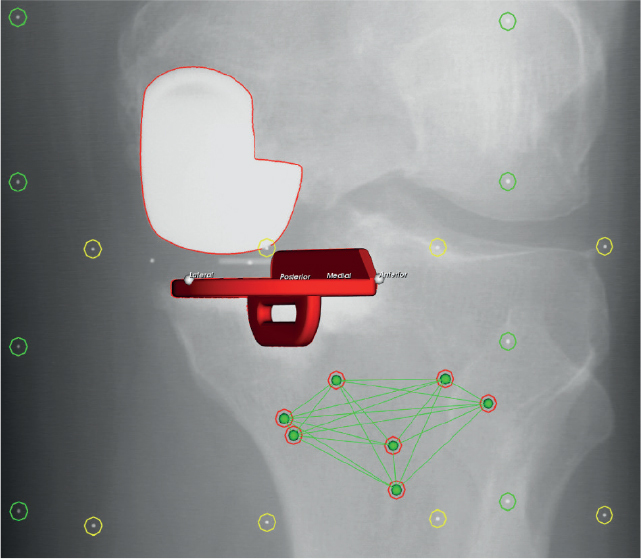
Computer-aided design (CAD) model of the tibial component with 4 virtual feature points (anterior, posterior, medial, and lateral) for evaluation of tibial component migration. Left knee, single view from RSA image.

Supine stereoradiographs were obtained on the first postoperative day (baseline) and at 6 weeks, 3 and 6 months, and 1 and 2 years. Computer-aided design (CAD) models of the tibial components were coded with 4 virtual feature points (anterior, posterior, medial, and lateral) for evaluation of tibial component migration (RSAcore, Leiden, the Netherlands). The stereographs were analyzed with Model-Based RSA (Version 4.01, RSAcore, Leiden, the Netherlands) [13]. Tibial component migration was measured with the tantalum markers in the proximal tibia as reference [14]. Tibial component migration was presented in a right-handed coordinate system, and data was normalized to presentation for the right knee, as translations along the x-axis (+medial/–lateral), y-axis (+lift-off/–subsidence), and z-axis (+anterior/–posterior) for all 4 feature points (Figure 5). For each of the 4 feature points, the total translation (TT) was determined as the Euclidian distance between the feature points present and previous position (TT = √(x^2^ + y^2^ + z^2^)). Continuous migration was defined as TT ≥ 0.2 mm between 1- and 2-year follow-up (stabilization phase) [15]. Precision was calculated as coefficient of repeatability (CR) using double examinations at 6 months (ISO 2013). The postoperative stereoradiograph was used as the reference in migration analysis of the double examinations (complete patient/system reposition) and the precision was presented as coefficient of repeatability (CR). Precision of RSA is presented in Table 1 (see Appendix).

**Table 1:** Double-examination measurement error of tibial component radiostereometric analyses (4 feature points combined)

Factor	Translations, mm			
	x-axis	y-axis	z-axis	TT^a^
Mean difference**^b^**	–0.010	–0.005	–0.026	–0.002
SD of difference**^c^**	0.078	0.065	0.115	0.087
CR**^d^**	0.152	0.127	0.225	0.171

a TT (total translation) calculated using TT = √(x^2^ + y^2^ + z^2^).

b Represents the systematic measurement error.

c Represents the random variation within the measurement comparing the double examinations. SD = standard deviation.

d CR (coefficient of repeatability) = 1.96 × SD represents the precision on individual measurements.

### Sample size

A generally accepted threshold for migration is the difference in total implant migration between 12 and 24 months > 0.2 mm [4]. To detect a 0.2 mm difference in total implant migration, 22 patients (knees) are needed in each group (power 90%, alpha 0.05, SD 0.2 mm) [16]. To anticipate dropouts, 25 patients (knees) were included in each group.

### Statistics

Normality of data distribution was evaluated using quantile-quantile plots. Normally distributed continuous data was compared using Student’s t-test. Categorical data was tested with s chi-square test. Mixed model analysis was used for tibial component migration (primary effect measure) of the 4 feature points as well as for TT in 3D. The migration data is reported as predicted means (95% confidence intervals [CI]). Multiple linear regression analyses were used to test the association between the investigated preoperative histomorphometric, µCT parameters and migration of the posterior feature point at 6 weeks and 2 years, crude and adjusted for age, BMI, and sex. Pearson’s rank correlation coefficient was used to test pairwise correlations between continuous variables. Mukaka’s interpretation criteria of correlation coefficient were used (0.00–0.30 = negligible correlation, 0.30–0.50 = low correlation, 0.50–0.70 = moderate correlation, 0.70–0.90 = high correlation, and 0.90–1.00 = very high correlation) [17]. STATA (v. 16.1, StataCorp LLC, College Station, TX, USA) was used for statistical analyses. The level of significance was 5%.

### Ethics, registration, funding, and disclosures

The current study was a subset of a 3-armed randomized controlled trial that was conducted in adherence with the Helsinki II declaration. Approvals were obtained from the local ethics committee (M-20070258; d. 15/01/2008) and the Data Protection Agency (2008-41-2104; d. 28/03/2008). The study was registered on ClinicalTrials.gov (NCT00679120). Zimmer Biomet funded the radiostereometric analyses, the Messerschmidt Foundation funded the µCT scans, and the Danish Rheumatology Association funded the histological evaluation. The µCT scanner was donated by the VELUX foundation. The funding bodies had no influence on the interpretation of results or writing of the manuscript. All authors report no conflict of interests. Complete disclosure of interest forms according to ICMJE are available on the article page, doi: 10.2340/17453674.2024.39917

## Results

Originally 80 patients were randomized to 3 study groups with medial UKR. In this secondary analysis we study 2 groups only, one group comprising 26 cemented components and another group comprising 25 cementless components (Figure 1). No patients were lost to follow-up or excluded, consequently all were analyzed to 2 years’ follow-up.

### Study population characteristics

There was no difference in demographic baseline characteristics between the cemented and the cementless groups (Table 2).

**Table 2:** Patient demographics at baseline. Values are mean (SD) or count

Factor	Cemented (n = 26)	Cementless (n = 25)
Age, years	64 (9)	65 (10)
Height, cm	175 (9)	173 (9)
Weight, kg	95 (16)	92 (14)
BMI	31 (6)	31 (4)
Side, right/left, n	17/9	11/14
Men/women, n	18/8	18/7

### Histomorphometry

**Figure 6:**
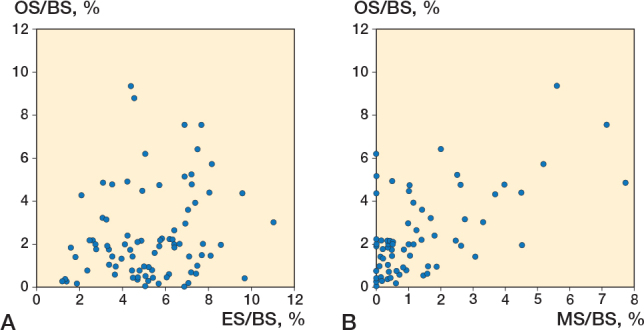
A. Relationship between the percentage of bone surface covered in osteoid (osteoid surface [OS]/bone surface [BS]) and the percentage of bone surface occupied by resorption cavities (eroded surface [ES]/BS). B. Relationship between the percentage of bone surface covered in osteoid (OS/BS) and the percentage of bone surface that displays a tetracycline label reflecting active mineralization (mineralizing surface [MS]/BS).

ES/BS was 2 twice as high as OS/BS in both the cemented and the cementless group (Table 3, Figure 6). MS/OS was 60% (SD 57) in the cemented group and 56% (SD 38) in the cementless group. The number of tetracycline double-labelled bone surfaces was too low to reliably estimate mineral apposition rate (MAR) and hence BFR/BS. Therefore, ES/BS, OS/BS, and MS/BS were used as proxy markers for bone resorption and formation. For the cemented group, ES/BS, OS/BS, and MS/BS showed low to negligible correlation with subsidence of the posterior feature point and TT at all follow-ups. For the cementless group, ES/BS and OS/BS had a negligible correlation, while MS/BS and MS/OS showed a moderate correlation of (r = –0.55, P = 0.03 and r = –0.51, P = 0.04, respectively) with subsidence of the posterior feature point and TT at all follow-ups. The subsidence of the other feature points was not correlated to ES/BS, OS/BS, MS/BS, or MS/OS for either the cemented or the cementless group (r < 0.39, P = 0.07). The crude and adjusted linear regression analysis (age, BMI, and sex) showed no relationship between migration of the posterior feature point and the investigated histomorphometric and µCT parameters (Tables 4 and 5, see Appendix).

**Table 3:** Preoperative histomorphometry. Values are mean (SD)

Factor	Cemented (n = 26)	Cementless (n = 25)
Eroded surface (ES/BS), %	4.6 (1.7)	4.7 (2.9)
Osteoid surface (OS/BS), %	2.1 (1.9)	2.3 (2.1)
Mineralizing surface (MS/BS), %	1.3 (1.5)	1.5 (1.3)
Mineralizing surface (MS/OS), %	60 (57)	56 (38)
Single-labeled surface (sLS/BS), %	1.1 (1.8)	1.1 (1.2)
Double-labeled surface (dLS/BS), %	0.7 (1)	0.9 (0.9)

BS = bone surface.

**Table 4:** Multiple linear regression models with preoperative MS/BS, OS/BS, ES/BS, and vBMD as predictors of 2-year tibial component total translation of the posterior feature point. All preoperative variables were analyzed as individual multiple linear regression analyses adjusted for preoperative age, sex, and BMI

Factor	Coefficient (SE)	CI
Mineralizing surface (MS/BS)	0.08 (0.07)	–0.07 to 0.23
Osteoid surface (OS/BS)	0.00 (0.04)	–0.08 to 0.08
Eroded surface (ES/BS)	–0.05 (0.03)	–0.12 to 0.01
vBMD	0.00 (0.00)	0.00 to 0.00

SE = standard error; CI = 95% confidence interval;

BS = bone surface; vBMD = volumetric bone mineral density.

**Table 5:** Correlations between posterior subsidence and histomorphometric and µCT parameters, given as Pearson correlation coefficient and by linear regression: correlation for subsidence in relation to the posterior feature point (y-axis) at 6 weeks is shown

	Crude coefficient						Adjusted coefficient (age, BMI, sex)			
	Pearson correlation		Linear regression (CI)				Linear regression (CI)			
	Cemented (n = 26)	Cementless (n = 25)	Cemented (n = 26)		Cementless (n = 25)		Cemented (n = 26)		Cementless (n = 25)	
vBMD1	–0.04	–0.23	–0.000061	(–0.0014 to 0.0013)	–0.0012	(–0.0030 to 0.00071)	–0.00039	(–0.0018 to 0.0011)	–0.0012	(–0.0032 to 0.00068)
vBMD2	–0.39	–0.21	–0.00075	(–0.0022 to 0.00075)	–0.00085	(–0.0023 to 0.00059)	–0.0011	(–0.0027 to 0.00052)	–0.0010	(0.0025 to 0.00042)
vBMD3	–0.15	–0.06	–0.00028	(–0.0018 to 0.0012)	–0.00028	(–0.0019 to 0.0014)	–0.00034	(–0.0019 to 0.0012)	–0.00045	(–0.0022 to 0.0013)
ES/BS	0.14	0.15	0.011	(–0.077 to 0.099)	0.026	(–0.027 to 0.080)	–0.0040	(–0.091 to 0.083)	0.41	(–0.014 to 0.096)
OS/BS	0.02	–0.08	0.0015	(–0.080 to 0.083)	–0.020	(–0.096 to 0.057)	–0.020	(–0.10 to 0.065)	0.00055	(–0.080 to 0.081)
MS/BS	–0.32	–0.55	–0.030	(–0.15 to 0.087)	–0.25	(–0.40 to –0.11)	–0.050	(–0.18 to 0.081)	–0.22	(–0.38 to –0.51)
MS/OS	–0.28	–0.51	–0.00071	(–0.0039 to 0.0025)	–0.0081	(–0.013 to –0.0029)	–0.00080	(–0.0040 to 0.0024)	–0.0086	(–0.014 to –0.0031)
Tb.Th1	0.01	–0.32	0.048	(–2.7 to 2.8)	–4.04	(–8.6 to 0.51)	–0.12	(–3.0 to 2.8)	–4.27	(–8.9 to 0.41)
Tb.Th2	–0.26	–0.11	–1.2	(–4.9 to 2.5)	–1.1	(–4.5 to 2.4)	–1.21	(–5.1 to 2.6)	–1.68	(–5.2 to 1.9)
Tb.Th3	0.07	0.05	0.29	(–3.4 to 4.0)	0.51	(–3.6 to 4.6)	0.42	(–3.5 to 4.3)	0.37	(–3.9 to 4.6)
Tb.Sp.1	0.04	0.04	0.056	(–1.1 to 1.2)	0.13	(–1.2 to 1.5)	0.40	(–0.88 to 1.7)	0.23	(–1.2 to 1.6)
Tb.Sp2	0.43	0.33	0.86	(–0.69 to 2.4)	1.26	(–0.079 to 2.6)	1.3	(–0.38 to 2.9)	1.2	(–0.13 to 2.5)
Tb.Sp3	0.32	0.06	0.46	(–0.70 to 1.6)	0.24	(–1.2 to 1.7)	0.48	(–0.71 to 1.7)	0.35	(–1.2 to 1.9)
BV/TV1	–0.06	–0.06	–0.085	(–1.2 to 1.1)	–1.13	(–2.7 to 0.44)	–0.38	(–1.63 to 0.88)	–1.22	(–2.8 to 0.39)
BV/TV2	–0.41	–0.41	–0.65	(–1.9 to 0.58)	–0.80	(–2.0 to 0.39)	–0.91	(–2.2 to 0.41)	–0.92	(–2.1 to 0.28)
BV/TV3	–0.17	–0.17	–0.26	(–1.6 to 1.0)	–0.35	(–1.8 to 1.1)	–0.30	(–1.6 to 1.0)	–0.51	(–2.0 to 0.98)
SMI1	0.14	0.14	0.026	(–0.12 to 0.17)	0.19	(–0.028 to 0.40)	0.059	(–0.096 to 0.22)	0.20	(–0.018 to 0.42)
SMI2	0.40	0.40	0.066	(–0.063 to 0.19)	0.080	(–0.060 to 0.22)	0.092	(–0.050 to 0.23)	0.086	(–0.055 to 0.23)
SMI3	0.13	0.13	0.024	(–0.12 to 0.17)	0.053	(–0.12 to 0.23)	0.035	(–0.12 to 0.19)	0.081	(–0.11 to 0.27)

For each VOI (1–3):

vBMD = volumetric bone mineral density; ES/BS = preoperative eroded surface; OS/BS = osteoid surface; MS/BS = mineralizing surface; MS/OS = ratio of mineralizing surface to osteoid surfaces; Tb.Th = trabecular thickness, i.e., mean thickness of the trabeculae; Tb.Sp = trabecular separation, i.e., mean distance between the trabeculae; BV/TV = trabecular bone volume fraction; SMI = structure model index.

Coefficients in linear regression: the coefficient for the relationship between each of the investigated histomorphometric and µCT parameters and the migration of the knee prosthesis on the y-axis to 6 weeks for cemented and cementless knee prosthesis given as crude estimates and adjusted for age, BMI, and sex.

### µCT

In all VOIs, preoperative vBMD showed low or negligible correlation to age, BMI, and sex. In VOI-3, preoperative vBMD showed negligible correlation to subsidence of the posterior feature point at 6 weeks’ follow-up in the cemented group (r = –0.15, P = 0.5) and in the cementless group (r = –0.06, P = 0.8). Neither of the investigated microstructural parameters was correlated to tibial component subsidence (Table 4 in Appendix and Table 6). There was no difference in vBMD in any of the 3 VOIs, BMI, age, MS/BS, ES/BS, or sex between the patients.

**Table 6:** Preoperative µCT parameters. Values are mean (SD)

Factor	Cemented (n = 26)	Cementless (n = 25)
VOI-1		
vBMD, mg/cm^3^	345 (87)	309 (72)
Tb.Th, *µ*m	206 (41)	192 (29)
Tb.Sp, *µ*m	561 (101)	610 (103)
BV/TV, %	32 (10)	28 (9)
SMI	0.1 (0.8)	0.4 (0.6)
VOI-2		
vBMD, mg/cm^3^	445 (75)	450 (92)
Tb.Th, *µ*m	233 (31)	244 (39)
Tb.Sp, *µ*m	445 (71)	476 (96)
BV/TV, %	44 (9)	45 (11)
SMI	–1.0 (0.9)	–1.0 (0.9)
VOI-3		
vBMD, mg/cm^3^	310 (77)	300 (83)
Tb.Th, *µ*m	191 (32)	200 (34)
Tb.Sp, *µ*m	560 (99)	603 (93)
BV/TV, %	29 (9)	28 (10)
SMI	0.4 (0.8)	0.6 (0.8)

VOI: volume of interest,

vBMD: volumetric bone mineral density,

Tb.Th: trabecular thickness, i.e., mean thickness of the trabeculae,

Tb.Sp: trabecular separation, i.e., mean distance between the trabeculae,

BV/TV: trabecular bone volume fraction,

SMI: structure model index.

### Radiostereometric analysis

**Figure 7:**
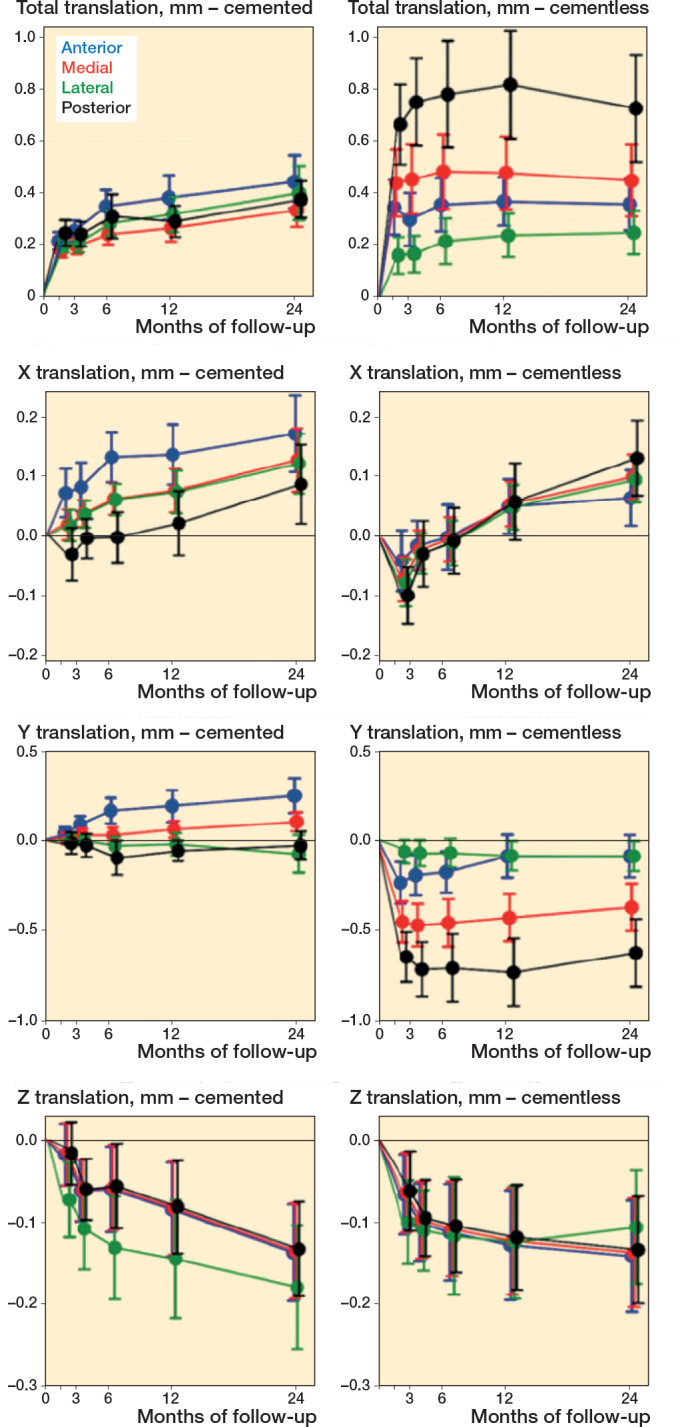
TT-translation, X translation (+ medial/– lateral), Y translation (+ lift-off/– subsidence), and Z translation (+ anterior/– posterior) in mm during follow-up in the 4 feature points.

Tibial component migration of the 4 feature points is presented in Table 7 and Figure 7.

**Table 7:** Translation of the tibial components as mean mm (CI) along the x, y, and z-axis measured with RSA at 6 weeks, 3 and 6 months, and 1 and 2 years after surgery

Axis Feature point	Cemented (n = 26)	Cementless (n = 25)	Mean difference
x-axis translation, mm (+ medial/– lateral)			
Anterior point			
6 weeks	0.07 (–0.00 to 0.14)	–0.04 (–0.11 to 0.03)	–0.11 (–0.21 to –0.01)
3 months	0.08 (0.01 to 0.15)	–0.02 (–0.09 to 0.05)	–0.10 (–0.20 to 0.00)
6 months	0.13 (0.06 to 0.20)	–0.00 (–0.08 to 0.067	–0.13 (–0.23 to –0.03)
1 year	0.13 (0.06 to 0.20)	0.05 (–0.02 to 0.12)	–0.08 (–0.18 to 0.02)
2 years	0.18 (0.11 to 0.25)	0.07 (–0.00 to 0.14)	–0.11 (–0.22 to –0.01)
Medial point			
6 weeks	0.03 (–0.02 to 0.08)	–0.08 (–0.14 to –0.03)	–0.11 (–0.19 to –0.04)
3 months	0.04 (–0.01 to 0.09)	–0.03 (–0.08 to 0.02)	–0.07 (–0.14 to 0.00)
6 months	0.07 (0.02 to 0.12)	–0.01 (–0.06 to 0.04)	–0.08 (–0.15 to –0.01)
1 year	0.08 (0.03 to 0.13)	0.05 (0.00 to 0.10)	–0.03 (–0.10 to 0.04)
2 years	0.14 (0.09 to 0.19)	0.01 (0.05 to 0.15)	–0.04 (–0.11 to 0.03)
Lateral point			
6 weeks	0.03 (–0.02 to 0.08)	–0.09 (–0.14 to –0.04)	–0.12 (–0.19 to –0.05)
3 months	0.04 (–0.01 to 0.09)	–0.03 (–0.09 to 0.02)	–0.08 (–0.15 to –0.01)
6 months	0.07 (0.02 to 0.12)	–0.02 (–0.07 to 0.04)	–0.09 (–0.16 to –0.01)
1 year	0.08 (0.03 to 0.13)	0.05 (–0.01 to 0.10)	–0.04 (–0.11 to 0.04)
2 years	0.14 (0.08 to 0.19)	0.10 (0.04 to 0.15)	–0.04 (–0.11 to 0.03)
Posterior point			
6 weeks	–0.01 (–0.08 to 0.07)	–0.12 (–0.20 to –0.04)	–0.11 (–0.22 to –0.01)
3 months	0.01 (–0.06 to 0.09)	–0.03 (–0.11 to 0.05)	–0.04 (–0.15 to 0.06)
6 months	0.02 (–0.06 to 0.09)	–0.01 (–0.09 to 0.06)	–0.03 (–0.14 to 0.08)
1 year	0.04 (–0.04 to 0.12)	0.05 (–0.02 to 0.13)	0.01 (–0.10 to 0.12)
2 years	0.10 (0.03 to 0.18)	0.13 (0.05 to 0.21)	0.03 (–0.08 to 0.14)
y-axis translation, mm (+ lift-off/– subsidence)			
Anterior point			
6 weeks	0.05 (–0.09 to 0.19)	–0.24 (–0.38 to –0.09)	–0.29 (–0.48 to –0.09)
3 months	0.10 (–0.04 to 0.24)	–0.19 (–0.33 to –0.05)	–0.29 (–0.49 to –0.09)
6 months	0.18 (0.04 to 0.32)	–0.17 (–0.31 to –0.03)	–0.35 (–0.55 to –0.15)
1 year	0.22 (0.08 to 0.36)	–0.09 (–0.23 to 0.06)	–0.31 (–0.51 to –0.11)
2 years	0.27 (0.13 to 0.41)	–0.09 (–0.23 to 0.06)	–0.36 (–0.56 to –0.16)
Medial point			
6 weeks	0.04 (–0.10 to 0.17)	–0.46 (–0.59 to –0.33)	–0.50 (–0.68 to –0.31)
3 months	0.05 (–0.08 to 0.18)	–0.48 (–0.61 to –0.34)	–0.53 (–0.71 to –0.34)
6 months	0.05 (–0.08 to 0.18)	–0.46 (–0.59 to –0.33)	–0.51 (–0.70 to –0.33)
1 year	0.09 (–0.04 to 0.22)	–0.43 (–0.57 to –0.30)	–0.52 (–0.71 to –0.33)
2 years	0.12 (–0.01 to 0.25)	–0.37 (–0.50 to –0.24)	–0.49 (–0.68 to –0.31)
Lateral point			
6 weeks	0.01 (–0.09 to 0.11)	–0.05 (–0.15 to 0.05)	–0.06 (–0.20 to 0.08)
3 months	0.00 (–0.10 to 0.10)	–0.06 (–0.17 to 0.04)	–0.07 (–0.21 to 0.08)
6 months	–0.03 (–0.13 to 0.07)	–0.06 (–0.16 to 0.04)	–0.03 (–0.17 to 0.11)
1 year	–0.02 (–0.12 to 0.08)	–0.08 (–0.18 to 0.02)	–0.06 (–0.20 to 0.08)
2 years	–0.08 (–0.19 to 0.02)	–0.09 (–0.19 to 0.01)	–0.01 (–0.15 to 0.14)
Posterior point			
6 weeks	0.02 (–0.16 to 0.20)	–0.66 (–0.84 to –0.48)	–0.68 (–0.94 to –0.42)
3 months	0.01 (–0.17 to 0.19)	–0.73 (–0.91 to –0.55)	–0.74 (–0.99 to –0.48)
6 months	–0.07 (–0.25 to 0.11)	–0.71 (–0.90 to –0.53)	–0.65 (–0.90 to –0.39)
1 year	–0.04 (–0.22 to 0.14)	–0.74 (–0.92 to –0.55)	–0.70 (–0.96 to –0.44)
2 years	–0.01 (–0.20 to 0.17)	–0.62 (–0.81 to –0.44)	–0.61 (–0.87 to –0.35)
z-axis translation, mm (+ anterior/– posterior)			
Anterior point			
6 weeks	–0.02 (–0.09 to 0.06)	–0.07 (–0.15 to 0.01)	–0.05 (–0.16 to 0.06)
3 months	–0.07 (–0.14 to 0.01)	–0.11 (–0.19 to –0.03)	–0.04 (–0.15 to 0.07)
6 months	–0.07 (–0.14 to 0.01)	–0.13 (–0.20 to –0.05)	–0.06 (–0.17 to 0.05)
1 year	–0.08 (–0.16 to –0.01)	–0.13 (–0.21 to –0.05)	–0.05 (–0.16 to 0.06)
2 years	–0.14 (–0.21 to –0.06)	–0.15 (–0.23 to –0.07)	–0.01 (–0.12 to 0.01)
Medial point			
6 weeks	–0.02 (–0.09 to 0.06)	–0.07 (–0.14 to 0.01)	–0.05 (–0.16 to 0.06)
3 months	–0.07 (–0.14 to 0.01)	–0.11 (–0.18 to –0.03)	–0.04 (–0.15 to 0.07)
6 months	–0.06 (–0.14 to 0.01)	–0.12 (–0.20 to –0.04)	–0.06 (–0.17 to 0.05)
1 year	–0.08 (–0.16 to –0.01)	–0.13 (–0.20 to –0.05)	–0.05 (–0.15 to 0.06)
2 years	–0.13 (–0.21 to –0.06)	–0.14 (–0.22 to –0.07)	–0.01 (–0.12 to 0.10)
Lateral point			
6 weeks	–0.06 (–0.15 to 0.03)	–0.11 (–0.21 to –0.02)	–0.06 (–0.19 to 0.07)
3 months	–0.10 (–0.19 to –0.01)	–0.12 (–0.21 to –0.03)	–0.01 (–0.14 to 0.11)
6 months	–0.12 (–0.21 to –0.03)	–0.13 (–0.22 to –0.04)	–0.01 (–0.14 to 0.12)
1 year	–0.13 (–0.22 to –0.04)	–0.13 (–0.22 to –0.04)	–0.00 (–0.13 to 0.13)
2 years	–0.18 (–0.27 to –0.09)	–0.12 (–0.21 to –0.02)	0.06 (–0.07 to 0.19)
Posterior point			
6 weeks	–0.02 (–0.09 to 0.06)	–0.07 (–0.14 to 0.01)	–0.05 (–0.16 to 0.06)
3 months	–0.07 (–0.14 to 0.01)	–0.10 (–0.18 to –0.03)	–0.04 (–0.14 to 0.07)
6 months	–0.06 (–0.14 to 0.01)	–0.12 (–0.19 to –0.04)	–0.06 (–0.16 to 0.05)
1 year	–0.08 (–0.16 to –0.01)	–0.12 (–0.20 to –0.05)	–0.04 (–0.15 to 0.07)
2 years	–0.13 (–0.21 to –0.06)	–0.14 (–0.22 to –0.06)	–0.01 (–0.12 to 0.10)
TT-translation, mm			
Anterior point			
6 weeks	0.22 (0.11 to 0.33)	0.46 (0.35 to 0.57)	0.24 (0.08 to 0.40)
3 months	0.26 (0.15 to 0.37)	0.41 (0.30 to 0.53)	0.15 (–0.01 to 0.31)
6 months	0.36 (0.24 to 0.47)	0.47 (0.35 to 0.58)	0.11 (–0.05 to 0.27)
1 year	0.40 (0.29 to 0.52)	0.48 (0.36 to 0.59)	0.07 (–0.09 to 0.23)
2 years	0.46 (0.35 to 0.57)	0.46 (0.35 to 0.58)	0.01 (–0.16 to 0.17)
Medial point			
6 weeks	0.17 (0.05 to 0.29)	0.54 (0.41 to 0.67)	0.37 (0.19 to 0.55)
3 months	0.19 (0.06 to 0.31)	0.55 (0.42 to 0.67)	0.36 (0.18 to 0.54)
6 months	0.23 (0.11 to 0.36)	0.56 (0.44 to 0.69)	0.33 (0.15 to 0.51)
1 year	0.27 (0.14 to 0.39)	0.56 (0.44 to 0.69)	0.30 (0.12 to 0.47)
2 years	0.34 (0.21 to 0.46)	0.53 (0.40 to 0.66)	0.19 (0.016 to 0.37)
Lateral point			
6 weeks	0.19 (0.10 to 0.28)	0.31 (0.22 to 0.41)	0.12 (–0.01 to 0.26)
3 months	0.21 (0.12 to 0.30)	0.30 (0.21 to 0.40)	0.09 (–0.04 to 0.23)
6 months	0.28 (0.19 to 0.38)	0.35 (0.26 to 0.45)	0.07 (–0.07 to 0.20)
1 year	0.33 (0.23 to 0.42)	0.36 (0.27 to 0.46)	0.04 (–0.10 to 0.17)
2 years	0.42 (0.32 to 0.51)	0.37 (0.27 to 0.46)	–0.05 (–0.19 to 0.08)
Posterior point			
6 weeks	0.22 (0.05 to 0.39)	0.74 (0.57 to 0.91)	0.52 (0.28 to 0.76)
3 months	0.22 (0.05 to 0.39)	0.80 (0.63 to 0.98)	0.58 (0.34 to 0.83)
6 months	0.29 (0.12 to 0.46)	0.82 (0.65 to 0.99)	0.53 (0.29 to 0.77)
1 year	0.29 (0.12 to 0.46)	0.85 (0.68 to 1.02)	0.56 (0.31 to 0.80)
2 years	0.38 (0.21 to 0.55)	0.76 (0.59 to 0.93)	0.38 (0.14 to 0.62)

At 2 years’ follow-up, for the posterior feature point, the cementless tibial components migrated more than the cemented (total translation 0.76 mm, CI 0.59–0.93 versus 0.38 mm, CI 0.21–0.55).

Tibial component subsidence was observed in the cementless group in the first 6 weeks at the medial 0.46 mm (CI 0.33–0.59) and posterior 0.66 mm (CI 0.48–0.84) feature points and from 3 months the cementless tibial components were stable. In the cemented group, there was no statistically significant tibial component migration of any feature point after the first 6 weeks. From 1 to 2 years’ follow-up, no cementless components but 4 cemented components showed continuous migration (defined as a > 0.2 mm threshold) [18].

After adjustment for age, BMI, and sex, the preoperative vBMD, ES/BS, OS/BS, and MS/BS did not show any association with 2-year tibial component TT in the posterior feature point Table 4 (see Appendix).

## Discussion

We investigated the association between RSA-measured tibial component migration at 2 years and baseline bone properties in terms of microstructure, vBMD, and bone turnover. The key finding was that preoperative microstructural and histological parameters had negligible predictive value for migration of both cemented and cementless tibial components. In addition, tibial component migration was mainly evident as subsidence of the posterior part of cementless components until 6 weeks, followed by stabilization from 3 months postoperatively. At 2-year follow-up, the cementless tibial components had migrated (total translation) 0.38 mm (CI 0.14– 0.62) more than the cemented components at the posterior feature point.

### Histomorphometry

Bone turnover was described by bone formation (OS/BS) and resorption (ES/BS) markers. In general, OS/BS is 2–3 times higher than ES/BS in steady-state, reflecting that bone formation usually takes longer than bone resorption [19]. However, in our study, OS/BS was approximately half the ES/BS, indicating that the proximal tibial bone was in a state of high bone resorption at the time of surgery. This discrepancy may be attributed to decreased use of the extremity due to pain or as a symptom of osteoarthritic pathology. A previous study on human hip osteoarthritis showed that increased bone turnover activity was associated with early osteoarthritis [20]. Osseointegration of cementless implants mandates bone formation for fixation [21]. However, we observed no correlation between resorptive bone surfaces (ES/BS) or osteoid surface (OS/BS) and cemented/cementless tibial component migration.

### µCT

DXA scanners provide density information as a projected areal BMD (aBMD), which is a 2-dimensional measure and therefore influenced by the size of the bone. In contrast, the CT technique provide volumetric BMD (vBMD), which is independent of the size of the bone and hence a true 3-dimensional density measurement. To our knowledge, vBMD has not previously been measured at the proximal tibia prior to insertion of UKR. Former studies have suggested that low preoperative proximal tibial aBMD may affect fixation of tibial components and lead to aseptic loosening [6,22]. We observed large vBMD variation between the anterior, central, and posterior VOIs, indicating that the tibial component is fixed on a surface of heterogeneous density. This is in accordance with previous studies [23]. However, the measured microstructural parameters were not correlated to component migration of either the cemented or the cementless tibial components. In support thereof, a recent study found that revision rates of cementless UKR were similar in patients with osteoporosis and in patients with normal aBMD [22].

Earlier studies found no association between preoperative systemic or proximal tibial aBMD and tibial component migration of cemented and cementless TKAs and UKRs until 2- to 5-year follow-up [6,24-26]. However, Li and Nilsson found an association between low preoperative proximal tibial aBMD and higher migration of cementless, but not of cemented, TKAs [27]. In addition, Petersen et al. found that maximal total point motion (MTPM) between 1 and 3 years showed a negative relation to aBMD (r = –0.47, P = 0.04) in uncemented TKAs. Hence, they observed less continuous migration in tibial components of knees with high preoperative tibial aBMD [28].

### Radiostereometric analysis

Initial implant subsidence has been suggested to be caused by an incomplete primary fixation with a time-lag until secondary osseous implant fixation [16]. Initially, we saw migration of cementless tibial components, mainly in the posterior part of the implant, which stabilized 3 months after surgery, consistent with the findings of Kendrik et al. [16]. In contrast, cemented tibial components had little or no subsidence, as also reported by Ryd et al. [4]. In addition, Kendrik et al. observed more initial subsidence in cementless than in cemented medial tibial component UKR [16]. At the geometric center of the tibial component, they found a mean subsidence of 0.23 mm (SD 0.18) in the cementless group within the first 3 months. We measured migration of the anterior, medial, lateral, and posterior edge of the tibial component and found subsidence primarily in the posterior part of the prosthesis and a corresponding lift-off in the anterior part of the prosthesis. Continuous migration in the second postoperative year has been reported as a predictor of aseptic loosening [4]. However, a subsequent RSA study did not find any difference between cemented and cementless UKR subsidence of the tibial component in the second postoperative year [16]. Likewise, we found low mean migration for both cemented and cementless tibial components between 1- and 2-year follow-up, which indicate good fixation on a group basis [4,16,29]. However, 4 cemented tibial components migrated > 0.2 mm compared with none in the cementless group, which indicate a risk of early aseptic loosening in 4/26 (15%) of cemented versus 0/25 (0%) of cementless tibial components.

### Strengths and limitations

The strengths of the present study were the use of highly accurate methods in terms of RSA for prosthesis migration and µCT for determination of vBMD and bone microstructure. The µCT technique allows for separation of cortical and trabecular bone and determination of vBMD. In contrast, aBMD determined by DXA is an area density, which is influenced by the geometry of the scanned object and other features such as exostoses and the condensed subchondral bone plate of osteoarthritic joints. Furthermore, µCT uses direct methods for determination of bone microstructure, while conventional histology uses model assumptions [12].

Limitations include a small number of patients per group, yet RSA is highly precise and the number is sufficient for evaluation of tibial component migration on group level [14]. We assessed the preoperative bone structure and bone turnover in a “mirror” of the bone surface where the tibial component was fixed. Potentially, there could have been differences in the “true” bone fixation surface. Furthermore, it is unknown whether the bone turnover changes after the surgical trauma.

### Conclusion

Tibial component migration 2 years after cemented or cementless medial UKR was not associated with baseline bone density, bone turnover, or microstructure. Our findings suggest that there is no need to consider age, sex, bodyweight, bone structure, or bone turnover when considering the indication for medial UKR. However, our findings cannot be extrapolated to obvious macroscopic abnormalities, e.g., bone defects and cysts.

## Notes

Study design: AO, FM, EMH, KS, and MS. Funding: KS and MS. Approvals and investigator: MS. Acquisition of data: AO, FM, JST, and MS. Analysis and interpretation of data: MAFB, AO, SBM, JST, EMH, and MS. Drafting of the manuscript: MAFB. Critical manuscript revision: MAFB, AO, FM, SBM, JST, EMH, KS, and MS.

Handling co-editors: Bart A Swierstra and Robin Christensen

*Acta* thanks Marc J Nieuwenhuijse and Tarja Anneli Soininvaara for help with peer review of this manuscript.
